# Enzymatic Conversion of Lignosulfonate into Wood Adhesives: A Next Step towards Fully Biobased Composite Materials

**DOI:** 10.3390/polym14020259

**Published:** 2022-01-08

**Authors:** Raphaela Hellmayr, Sabrina Bischof, Jasmin Wühl, Georg M. Guebitz, Gibson S. Nyanhongo, Nikolaus Schwaiger, Falk Liebner, Rupert Wimmer

**Affiliations:** 1 Institute of Wood Technology and Renewable Materials, University of Natural Resources and Life Sciences, Konrad Lorenz Straße 24, 3430 Tulln, Austria; raphaela.hellmayr@boku.ac.at; 2 Institute of Environmental Biotechnology, University of Natural Resources and Life Sciences, Konrad Lorenz Straße 20, 3430 Tulln, Austria; sabrina.bischof@boku.ac.at (S.B.); jasmin.wuehl@boku.ac.at (J.W.); guebitz@boku.ac.at (G.M.G.); g.nyanhongo@boku.ac.at (G.S.N.); 3 Austrian Centre for Industrial Biotechnology (ACIB), Konrad Lorenz Strasse 20, 3430 Tulln, Austria; 4 Sappi Papier Holding GmbH, Brucker Straße 21, 8101 Gratkorn, Austria; nikolaus.schwaiger@sappi.com; 5 Institute of Chemistry of Renewable Resources, University of Natural Resources and Life Sciences, Konrad Lorenz Straße 24, 3430 Tulln, Austria; falk.liebner@boku.ac.at

**Keywords:** laccase, lignosulfonate, bio-based adhesive, tensile-shear strength, wood adhesive, biotechnology

## Abstract

This study investigates the effect of the enzymatic polymerization of lignosulfonate for the formulation of a lignosulfonate-based adhesive. For this, beech lamellas were glued together and tested according to the EN 302-1 standard. The results showed that the laccase-polymerized lignosulfonate-based wood adhesives (LS-p) had similar mechanical properties as a standard carpenter’s glue (PVAc-based D3 class white glue), as no significant difference in tensile shear strength between these two adhesive types was found. However, carpenter’s glue showed almost 100% wood failure, while with the lignosulfonate-based wood glue, the samples failed, mainly in the glueline. Pre-polymerization of LS-p is the most critical factor to achieve the required viscosity, which is also connected to the wetting properties and the resulting tensile shear strength. The longer the pre-polymerization, the higher the viscosity of the LS-p adhesive, with the tensile shear strength reaching a plateau. The presented data show the potential of using enzymatically pre-polymerized lignosulfonate as a well-performing wood adhesive. Further development and optimization of the pre-polymerization process is required, which is also important to push towards upscaling and practical applications.

## 1. Introduction

Sulfite pulping in Europe has an annual production of 1.7 million tons [1] and affords a chemically modified type of lignin that is highly water soluble at all pH conditions due to its rich sulfonate group content [2,3]. Since lignin features further intriguing properties including amphiphilicity, aromaticity, antioxidant, and antimicrobial activity, the commercialization of lignosulfonates has been attracting the interest of both material scientists and pulp producers. Besides other applications such as concrete setting retarders, plasticizers, emulsifiers, dispersants, or binding agents [4], it is thus not surprising that lignin was already considered as a valuable and cheap source for bio-based adhesives in the former Eastern Bloc states [5]. Respective patents have been filed since the 1950s, proposing modified lignin as an adhesive to be used for, e.g., full-surface linoleum bonding on concrete floors [6], using paper board as an adhesion-promoting interlayer. Commercial lignosulfonate adhesives by the former company “Sächsische Klebstoffwerke” (e.g., Luxol D^®^, Bärenkleber-Blausiegel^®^, Bärenkleber Braunsiegel^®^) were widely used in the former German Democratic Republic until the early 1990s. Depending on the targeted application, lignosulfonates were blended with additives, such as dextrines for paper glues, or with Cr (VI) salts and gypsum for bonding of floor coverings [7]. Later, mixtures of lignosulfonates with kraft lignins were studied because the kraft process started to become the dominating wood pulping technology. Since the lignins obtained from kraft technology are poorly water-soluble except for strongly alkaline conditions, activation in terms of depolymerization and introduction of polar functional groups by phenol oxidases was tested. Respective mixtures of such as activated lignosulfonates and kraft lignins were proposed as a binder for inorganic particles, such as mineral wool [8] or—in combination with plant fibers—for clay–fiber composite insulation materials [9]. More recently, adhesives based on combinations of magnesium lignosulfonate with polysaccharides such as potato starch or monosaccharides such as sorbitol have been proposed [10]. Lignin could be also obtained by other wood processing methods such as, e.g., steam explosion of lignocellulosic biomass [11].

Gluing wood to produce solid wood furniture, plywood, laminated veneer lumber, oriented strand boards, particleboards, or fiberboards is a well-established and growing practice. Fitting the large range of targeted properties and applications (indoor, outdoor), a variety of adhesives is commercially available. This includes epoxide, acrylate, silane, urethane and poly(vinyl acetate) adhesives, next to the large family of resins relying on formaldehyde-based methylolation of phenol, resorcinol, urea, or melamine [12]. During the past decade, considerable efforts have been made to explore the possibility of co-utilizing lignin in the above synthetic adhesives, reasoned by its significantly lower price, its reactive phenolic base structure and three-dimensional glue network expanding capabilities. Recent activities are aiming at the development of entirely formaldehyde-free or even fully biobased wood adhesives [13]. Hence, lignin has increasingly moved into the spotlight of adhesive research, to be used for production of particleboards [13] or fiberboards [14]. The latter, for example, have been prepared by hot pressing mixed Norway spruce and Scots pine fiber mass, using 15 wt.% of commercial magnesium lignosulfonate as a binder [15]. While the obtained composite materials featured acceptable mechanical properties (above minimum requirements for type P2 particleboards according to European standard EN 312), the thickness swelling and water absorption were too high, at least for load-bearing applications [15]. This is indicative of either insufficient lignin crosslinking density, a high proportion of polar moieties remaining after bonding, and/or improper lignin molecular weight characteristics.

Guided by the desire to explore the potential of fully biobased adhesive systems and in attempt to implement essential principles of “green chemistry” [16] and waste-free “cradle-to-cradle” [17] philosophies, this paper investigates the pre-activation of lignin by phenol oxidases as a means towards enhanced reactivity. Laccases form a large family of multi-copper oxidases capable of catalysing one-electron oxidations of a variety of substrates, including polyphenols such as lignin. The liberated electrons are transferred to a copper cluster concomitantly causing a four-electron reduction of O_2_ to H_2_O while reactive intermediates are formed from the substrates that can subsequently stabilize via multiple pathways [18]. Interestingly, laccases of different origin can have contrastive biological functions [19]. While plant laccases catalyse the dehydropolymerization of lignols to build up the three-dimensional polymer lignin, fungal laccases have an adverse mode of action, i.e., catalysis of lignin degradation to reactive lower-molecular compounds (polyphenols, quinonid compounds), which enter then the various pathways towards the formation of soil humic substances [20,21]. In the limelight of current efforts centred around a better co-utilization of technical lignins, laccases have emerged as promising green catalysts capable of delignifying pulp [22] but—even more prominently—of synthesizing and modifying materials from different types of technical lignins, including lignosulfonate and kraft lignins [23,24,25]. Similar to native lignins, the reaction of laccases with technical lignins affords reactive key intermediates such as phenoxy radicals, which typically trigger lignin polymerization or/and crosslinking with other (functional) substrates. Both are of great interest for adhesives based on well-processible water-based lignosulfonate since solubility and hydrophilicity can be reversed in the course of the enzyme-catalysed modification. The desired effects are achieved as a result of both, the formation of C-C aryl-alkyl and aryl-aryl bonds, which can effectuate a considerable increase in molecular weight and the depletion of polar functional groups in favour of less polar moieties formed, such as ether linkages [26,27,28]. Besides their use as activating ingredient in lignin-based adhesives, laccases can be also used for in situ activation of any type of lignocellulosic biomass to make use of the intermediates formed for bonding and crosslinking purposes, respectively [29]. Targeting the preparation of fibreboards from enzymatically modified wood fibres, this has been extensively investigated for different types and quantities of laccases, types of lignocellulosic fibres, and a wide range of hot-pressing conditions [30,31]. A similar technology had been implemented into a pilot plant for the production of fibre boards from laccase-oxidized wood [32].

The existing literature shows that vivid research in the field of biobased wood adhesives is under way. Research on adhesives relying solely on lignin bonding capabilities, however, is still in its infancy. A two-step laccase activation/phenolation approach using various soda and kraft lignins has been reported targeting the development of an adhesive for wool floor covering [33]. Even though the adhesive reached good strength values, the required phenolation and addition of a plasticizer might not yet be the ideal solution. Based on existing literature, no published work dealing with pure enzymatic modified adhesive for solid wood has been found, which is therefore the aim of this work. Focusing on the above “cradle-to-cradle” idea [17] and the guidelines of the green chemistry philosophy [16], this paper investigates in more detail the effect of enzymatic polymerization of lignosulfonates on molecular weight characteristics, viscosity development, and their impact on tensile shear strength of glued solid wood samples after curing of the glueline.

## 2. Materials and Methods

All used chemicals were of analytical grade. The plasticizers glycerol and xylitol were obtained from Sigma-Aldrich (Steinheim, Germany). Potassium phosphate salts for preparing the buffers were purchased from Carl Roth (Karlsruhe, Germany), sodium hydroxide (NaOH) from Merck (Darmstadt, Germany), and poly(vinyl acetate) type D3 (PVAc) from Henkel (Düsseldorf, Germany). Laccase extracted from Myceliophthora thermophila (MTL) was purchased from Novozymes (Bagsvaerd, Denmark) and industrial softwood magnesium lignosulfonate (LS) was kindly provided by SAPPI (Gratkorn, Austria). Moreover, lignosulfonates were available at constant quality and amount and at relatively low cost available throughout the year. European beech (*Fagus sylvatica* L.) was used as a wood substrate in the gluing experiments. 

Laccase activity was determined spectrophotometrically as described elsewhere [34]. The method is based on the evaluation of the kinetics of the oxidation of 2,2′-azino-bis(3-ethylbenzothiazoline-6-sulfonic acid) diammonium salt (ABTS) to its cation radical. When laccases catalyze the oxidation of their substrates, corresponding free radicals are generated as products. ABTS is oxidized by laccases to the more stable and preferred state of the cation radical. This laccase-mediated oxidation process, which results in green colored water-based samples, can be monitored by following light adsorption at 420 nm, which was accomplished using a plate reader (Infinite M200, Tecan Group Ltd., Männedorf, Switzerland). Laccase activity was expressed in units (U), i.e., defined as the amount of enzyme necessary to convert 1 µmol substrate per minute. The activity of laccase was measured in 100 mM potassium phosphate buffer at pH 7. All experiments were carried out in triplicates.

**Figure 1:**
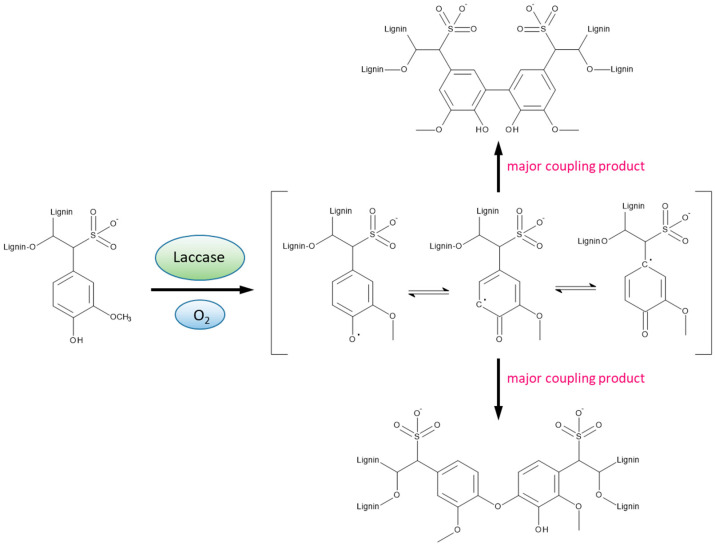
Laccase-mediated radical network expansion of lignosulfonates affording the two principal 5-5′ and 5-O-4′ coupling products.

Defined amounts of the air-dried, ground soft-wood lignosulfonates were dissolved in deionized water to give solutions with a solid content of 15% (*w*/*v*), if not indicated otherwise. Solutions were adjusted to pH 7 by adding aqueous sodium hydroxide. Aliquots of the solutions were transferred into 150 mL transparent durum bottles. In some experiments, 5% (*w*/*v*) glycerol or xylitol was added to these solutions as a plasticizer. The enzymatic modification was carried out at 20 °C in 150 mL transparent durum bottles, which were closed with screw caps, equipped with tight-fitting tubes on the lid allowing for continuous oxygen aeration at a flow rate of 10 mL min^−1^, as described in [28]. The enzymatic polymerization of lignosulfonate was started by introducing 1% *v*/*v* of laccase solution (10 mg/mL) related to the lignosulfonate solution, without adding any external mediator. Aliquots taken from the reaction mixture in certain time intervals were analysed with regard to viscosity to monitor the polymerization process. The laccase-mediated polymerization process via radical network expansion is resulting in two principle 5-5′ and 5-O-4′ coupling products [35] as displayed in Figure 1. After pre-set levels of viscosity (LS-p1: 100 mPa·s, LS-p2: 500 mPa·s, LS-p3: 1000 mPa·s) had been reached, corresponding to different degrees of pre-polymerization, the oxygen supply was stopped, and the respective batch was used immediately for bonding of the test beech-wood lamellas.

The rheological progression of the reaction was followed by viscosimetry using an Anton Paar Physica MCR 302 rheometer (Anton Paar GmbH, Graz, Austria) equipped with a CP50-1° cone (gap of 0.1 mm) and operated at a shear rate of 200 s^−1^ at 20 °C. 1.0 mL of the reaction mixture was withdrawn for each of the measurements and returned to the mixture afterwards.

The average molecular weight of magnesium lignosulfonate was determined by size exclusion chromatography at different stages of pre-polymerization as described elsewhere [36], using the following equipment: 1260 Infinity II series quaternary/binary pump, 1260 Infinity refractive index and diode array detectors, 1260 series auto sampler (all Agilent Technologies Inc., Santa Clara, CA, USA), magic angle laser light scattering (MALLS) HELEOS DAWN II detector (Wyatt Technologies Inc., Santa Barbara, CA, USA). The chromatographic system (Agilent Technologies Inc., Santa Clara, CA, USA) consisted of a PL aquagel-OH MIXED Guard pre-column (PL1149-1840, 8 μm, 7.5 × 50 mm) and a PL aquagel-OH MIXED H separation column (PL1549-5800, 4.6 × 250 mm, 8 μm) [28]. Data acquisition and analysis was accomplished using the Openlab Chemstation CDS (Agilent Technologies Inc., Santa Clara, CA, USA) and ASTRA 7 (Wyatt Technologies Inc., Santa Barbara, CA, USA) software packages. Bovine serum albumin (BSA) was used as standard for the normalization, band broadening and alignment of the MALLS detector. During each sampling, 0.1 mL was withdrawn from the reaction mixture and diluted with the mobile phase (50 mM NaNO_3_, 3 mM NaN_3_) to obtain a concentration of 1 mg mL^−1^. A 100 µL aliquot of this solution was used for SEC analysis. Bovine serum albumin (BSA) was used as standard for the normalization, band broadening and alignment of the MALLS detector.

Beech wood lamellas (120 × 500 mm) were equilibrated according to the EN 302-1 norm in a climate chamber (20 °C, 65% RH) for 10 days at least. The surface of these samples was smoothened using P100 grit sandpaper until a lamella thickness of 5 mm was reached. Then, the respective pre-polymerized lignosulfonate wood adhesive (180 g dry mass per square meter) was applied onto one side of each of the two lamellas to be glued, using a toothed spatula. After that, the lamellas were manually pressed together and the excess of adhesive squeezing out was removed. The glued specimens were then mounted into a hydraulic press (500 mm × 500 mm pressing area, Langzauner, Lambrechten, Austria) for curing of the glueline (0.8 MPa, room temperature, 12 h). Subsequently, the glued beech lamellas were stored in a climate chamber (20 °C, 65% relative humidity) until they were cut into single-lap shear samples (20 × 150 mm). Tensile shear testing was accomplished according to EN 302-1 using a Zwick/Roell Z020 universal testing machine (ZwickRoell GmbH & Co. KG, Ulm, Germany) operated at a testing speed of 1 mm min^−1^ until failure occurred. Wood failure was evaluated according to EN 302-1 as an estimation of the proportion of fractured surface covered with wood fibers. The force was recorded, and the tensile shear strength was calculated dividing the maximum force by the cross-sectional area. The glueline was further characterized by optical microscopy (Olympus DSX1000 Digital Microscope, Olympus Scientific Solutions Americas Inc., Waltham, MA, USA). Besides data analysis by descriptive statistics (box-and-whisker plots), analysis of variance (ANOVA, Bonferroni post hoc test) was performed to elucidate the impact of pre-polymerization intensity and plasticizer type (SPSS Statistics 26, International Business Machines Corporation (IBM^®^), Armonk, NY, USA). Unless otherwise stated, the tensile shear strength and viscosity testing were performed in 15 and 10 repetitions, respectively.

## 3. Results and Discussion

**Figure 2:**
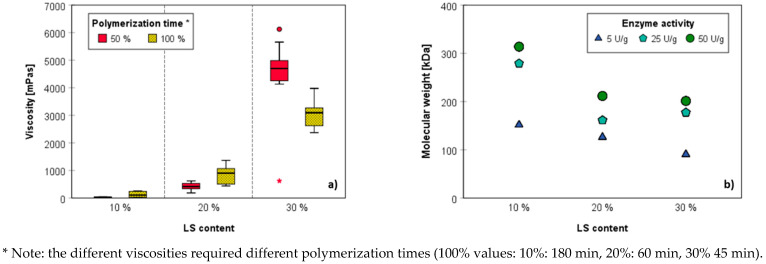
Impact of magnesium lignosulfonate (LS) content in laccase-mediated polymerization on viscosity (**a**) and weight average molecular weight (**b**).

Initial experiments investigating the effect of varying lignin contents on the viscosity, homogeneity, and applicability of the adhesive products obtained after laccase-mediated polymerization revealed that solid contents beyond 20 wt.% afforded products of inappropriate consistency. This is reflected to some extent also by the exponentially increasing viscosity (Figure 2a). Since magnesium lignosulfonate tends to clump and form aggregates in aqueous medium due to π-π stacking and the formation of extended hydration spheres (see below), the products obtained by polymerization of 30 wt.% solid content were considered inappropriate for gluing due to their inhomogeneous nature. Size exclusion chromatography, furthermore, showed that with increasing solid content, significant lower degrees of polymerization and weight average molecular weights were obtained (Figure 2b). In a compromise between viscosity and weight average molecular weight, it was decided to continue this study using magnesium lignosulfonate contents of 15 wt.%.

**Figure 3:**
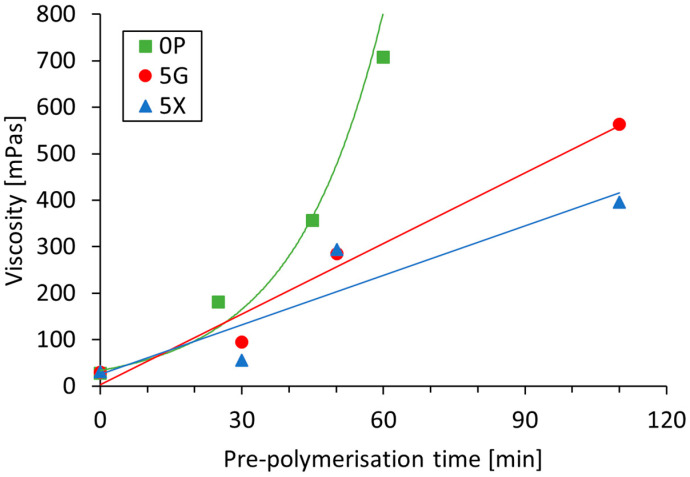
Viscosity development of LS solutions (15 wt.%, 20 °C) during laccase-mediated pre-polymerization in the absence (0P) and presence of 5 wt.% plasticizers (5G, 5X).

**Figure 4:**
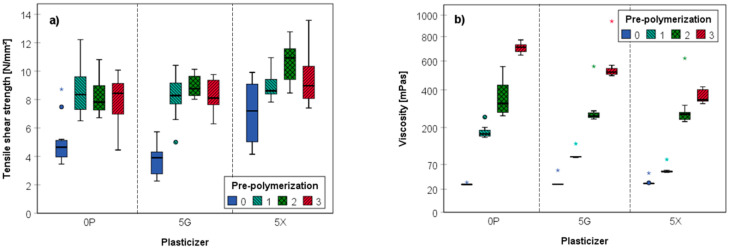
Tensile shear strength (N/mm^2^) (**a**) and viscosity (mPas) (**b**) of enzymatically pre-polymerized lignosulfonate-based adhesives (LS-p), without (0P) or with addition of 5 wt.% plasticizer (glycerol, 5G; xylitol, 5X). The respective parameters were recorded at four levels of pre-polymerization as defined by viscosity and differing, therefore, in polymerization time (for the individual polymerization times, see Table 1).

The effects of lignin viscosity, degree of pre-polymerization and addition of plasticizers on the tensile shear strength of beech-wood lamellas bonded with the obtained lignin glues is described in the following. First, it is to note that polymerization of the pure magnesium lignosulfonate seems to follow a different kinetics than is the case for mixtures that additionally contained 5 wt.% of plasticizers, i.e., glycerol (5G) or xylitol (5X). Polymerization of plasticizer-free aqueous solutions of magnesium lignosulfonate (15 wt.%) and laccase (unpolymerized LS-p) showed an exponential increase in viscosity from 27.69 mPa·s to 707.55 mPa·s after 60 min reaction time at 20 °C (*n* = 15; Figure 3 and Figure 4b). This is in good agreement with a previous study which also reported that both exponential change in viscosity and molecular weight upon enzymatic polymerization of industrial magnesium lignosulfonate is strongly dependent on the pH (fastest at pH 6) and temperature (fastest at 60 °C) [37]. Different from the pure lignosulfonate, the addition of plasticizers resulted in a more shallow and largely linear increase in viscosity (Figure 3), which was least pronounced when xylitol was used (Table 1). For both plasticizers tested, more than twice the reaction time of 0P was granted to reach reasonable viscosity values (563 mPa·s, 5G; 395 mPa·s; 5X) after 110 min.

The data shown in Figure 4a unambiguously reveal that pre-polymerization is essential to achieve reasonable glueline strength for the adhesives prepared from magnesium lignosulfonate. In unpolymerized form and containing no plasticizer, the mean tensile shear strength of bonded beech wood lamellas was 5.04 N∙mm^−2^ only. It should be noted that the term unpolymerized LS-p represents formulations that contain both the enzyme and respective additive but were not subjected to pre-polymerization. Depending on the type of plasticizer added, the average tensile shear strength of unpolymerized LS-p varied between 3.77 N∙mm^−2^ (5% glycerol) and 7.11 N∙mm^−2^ (5% xylitol), which is still below the lower limit (10 N∙mm^−2^) for wood adhesives (European standard EN 301). Intriguingly, significantly higher tensile shear strength values were obtained when the magnesium lignosulfonate was subjected to laccase-mediated pre-polymerization beforehand. Pre-polymerization of the parent lignin in the absence of plasticizers for 25 min, for example, increased the tensile shear strength from 5.04 to 8.22 N∙mm^−2^.

These values were achieved only after it had been ensured that sufficient amounts of oxygen were provided to the reaction mixture, which was accomplished by providing oxygen at a constant flow rate of 10 mL min^−1^. It is well known that oxygen is a mandatory co-substrate for laccase-mediated polymerization reactions and for unfolding the enzyme’s full activity [38]. This has been also confirmed in this study, as it turned out that the oxygen content of the liquid phase inside the reaction vessels dropped within seconds almost to baseline level, ceasing the polymerization until complete standstill [39].

However, even though a clear gain in tensile shear strength was observed upon pre-polymerization of LS, the data of Table 1 and Figure 4 clearly show that the values for the glued test specimen levelled off when the LS pre-polymerization had reached a critical value. This is at about 30 min polymerization time for the chosen lignin content of 15 wt.%. Similar to random lignols dehydropolymerization in lignin biosynthesis, pre-polymerization of LS starts with the generation of phenoxy radicals. The free electron formed can subsequently delocalize across the conjugated π system, creating a set of reactive sites that can undergo multiple reactions such as radical transfer or coupling to further substrates [40], which can be other lignin molecules or the plasticizers. During pressing, condensation of hydroxyl groups under the formation of ether bonds and the agglomeration of lignin by plasticization presumably also contribute to the final mechanical properties of the adhesive. It is safe to assume that once glued and cured, the glueline will maintain its bond strength, since laccase will be no longer active due to steric constraints and the virtual absence of oxygen.

Technologically, the application and spreading of the lignin adhesive strongly depends on the initial lignin concentration, the extent of pre-polymerization and—closely related—the final viscosity of the prepared adhesive. Products of high viscosity containing polymerized lignin are difficult to spread due to inhomogeneities by lump formation, as mentioned earlier. Therefore, the lignin-based adhesive was prepared at relatively low mass content (15 wt.%) affording a homogeneous adhesive, however, with less convenient applicability (low viscosity, fast water absorption) compared to white glue.

The tensile shear strength data of Table 1 clearly confirm that meaningful mechanical properties of the bonded beech wood lamellas can only be achieved after the magnesium lignosulfonate had been subjected to a laccase-mediated pre-polymerization.

The penetration of an adhesive into cavities and voids of the materials to be bonded—wood in this case—has a strong influence on the mechanical properties of the final composite [41]. While the extent of physical and chemical bonding within the bulk phase of the glueline decides about its resistance towards rupture and failure, molecular interlocking or cross-linking (e.g., covalent, ionic, van der Waals or hydrogen bonding; [42]) in voids of the neighboring near-surface layers is essential for dissipating forces across a wider zone. This measure typically increases the (desired) probability of wood failure, i.e., the situation when wood yields more easily than the glueline and is the reason why class D3 white glue contains diisocyanate as cross-linker. While, up to a certain lower limit, penetration is a prerequisite for connecting a robust glueline with the adjacent porous materials, over-penetration beyond an upper limit would be tantamount with the presence of an insufficient quantity of adhesive in the glueline [43]. Owing to its excellent water-solubility, the result of both the low molecular weight and high content of functional groups, magnesium lignosulfonate would be a poor adhesive. Therefore, the increase in molecular weight was required, which takes place at the expense of the number of polar groups. This, however, entails the formation of gelatinous products which are difficult to apply compared to common white glue. This was counteracted by choosing shorter polymerization times affording materials of higher homogeneity and better applicability.

The tensile shear strength measurements of beech wood assemblies cold press-bonded with enzymatically onward-polymerized magnesium lignosulfonate (LS-p) as a one-component adhesive showed surprisingly good results (Table 2). They were comparable to the mechanical properties of a commercial D3 class PVAc white glue, which additionally contains hexamethylene diisocyanate as a cross-linker and is a widely used wood adhesive. The wood assemblies used for mechanical testing were bonded with an aqueous dispersion/solution (15 wt.%) of LS-p either applied without plasticizer, or with 5 wt.% glycerol (5G) or xylitol (5X) and a viscosity of 500 mPas. Analysis of variance reveals that no significant differences exist between the tensile shear strength of PVAc (10.96 ± 1.07 N∙mm^−2^), plasticizer-free LS-p (10.57 ± 1.42 N∙mm^−2^), and LS-p bonded samples which contained 5% xylitol (10.54 ± 1.36 N∙mm^−2^). The lower minimum of 10 N∙mm^−2^ set by the EN 301 standard was reached by all adhesive types, except for LS-p that was amended with 5% glycerol. The decrease in tensile shear strength observed after adding the plasticizers is in good agreement with the results of a study investigating the influence of different plasticizers on the properties of films from an enzymatically polymerized lignosulfonate [29]: on the one hand, films containing 10 wt.% of glycerol showed very high strain at break, while the achieved strength values remained at a low level. On the other hand, the addition of 10 wt.% xylitol entailed higher strength values, while the strain at break was rather poor. Additionally, polymerized lignosulfonate films without plasticizers turned out to be brittle, with cracks already visible during the curing process at room temperature [29].

**Figure 5:**
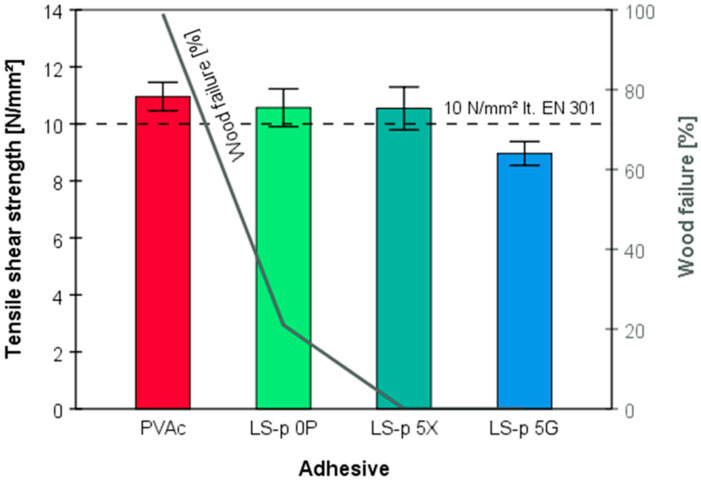
Tensile shear strength of PVAc-bonded single-lap shear samples, compared to samples glued with enzymatically polymerized lignosulfonate-based adhesive (LS-p) without plasticizer (0P), with containing 5% xylitol plasticizer (5X), or with containing glycerol (5G) plasticizer, *n* = 15 in each group.

The extent of wood failure tested for glued assemblies is a quality criterion for the adhesive used. It represents a value which assesses whether the glueline or the adjacent wooden parts fail first while applying increasing tensile shear force. Respective tests revealed that samples bonded with commercial PVAc (D3) showed almost 100% wood failure, while all enzymatically polymerized LS-p formulations (average viscosity 500 mPas) reached a significantly lower percentage of wood failure, if at all. Even though the tensile shear strength of LS-p and LS-5X were very similar and all above the EN 301 minimum limit (cf. Figure 5), only a low extent of wood failure (20%, *n* = 15) was observed for the plasticizer-free formulation.

These widely diverging results are obviously due to the different nature of the two glue systems. It should be noted that the reference common white glue represents a microemulsion of thermoplastic poly(vinyl acetate) latex particles in water, which is amended with a cross-linking agent (hexamethylene diisocyanate) to reach strength class D3. The lignin-based adhesives of this study, however, represent network polymers of limited melting capabilities. While the adhesive effect of white glue is a result of both physical merging of the PVAc microspheres by melting and cross-linking to hydroxyl groups of wood constituents under formation of ether and carbamate moieties [44,45], the lignin-based adhesives largely lack the capabilities of chemical cross-linking. Here, setting of an ordered state achieved by aggregation of lignin molecules in aqueous environment is supposed to be the driving force of adhesive strength development upon the evaporation and migration of water into adjacent cavities of wood under gluing conditions. It has been demonstrated for magnesium lignosulfonate that hydrophobic interactions [46] can entail relatively pronounced π-π stacking of phenolic moieties [47] leading to a hydrophobic core and hydrophilic outer surface of the aggregates formed. At high solute contents (e.g., 30 wt.%), these aggregates can form lumps which are even visible with the naked eye and prevent homogeneous spreading of the adhesive. Among commercial lignosulfonates, magnesium lignosulfonate is assumed to be particularly prone towards the formation of aggregates due to its capability to form large hydration spheres, supporting hydrogen bonding as a second bonding mechanism for lignosulfonate-lignosulfonate bonding [48]. It is worth mentioning that the formation of larger aggregates can be supported by pH lowering, by the addition of polyelectrolytes [49], or by increasing the temperature above 38 °C [50], all measures that have been shown to reduce both the zeta potential and the electrostatic repulsion in respective systems. It can be assumed that the abundance of sulfonate and carboxyl groups ensures good water-solubility, even for lignosulfonate aggregates formed. This is likely to promote diffusion of lignin into wood under pressing conditions, where interlocking of the randomly branched pre-polymerized polyelectrolyte lignin aggregates takes place.

The dominating occurrence of mechanical failure in the interphase between wood and adhesive instead in the bonded wooden parts has been also confirmed by optical microscopy. Respective micrographs of fracture surfaces originating from the sample that was glued with LS-p show single wood fibers sticking out of the surface, which is indicative of bonding failure.

**Figure 6:**
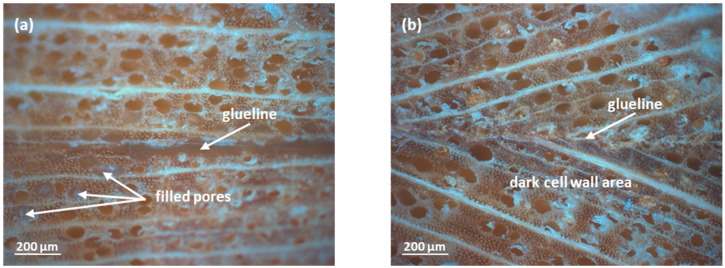
Reflected-light microscopic images of the wood samples glued with PVAc (**a**), and with LS-p without plasticizer (**b**). Notice the thicker glueline in (**a**), while (**b**) has a thin glueline, with lignosulfonates seemingly impregnating adjacent lumen and cell wall areas (“dark cell wall area”).

Glueline analysis by optical microscopy furthermore revealed distinct differences regarding the dimensions of the glueline. While for the specimen bonded with the PVAc-based adhesive a clearly visible glueline is formed with some material penetrating and filling adjacent larger cavities (Figure 6a), it was not the case when the enzymatically pre-polymerized lignin LS-p was used. Here, the glueline is barely visible, but dark zones next to the glueline indicate that the adhesive was mainly penetrating neighboring regions along the interfaces (Figure 6b). These differences are presumably caused by the different bonding mechanisms discussed above. While the thermoplastic PVAc latex particles (TG 18–45 °C) of white glue form a continuous phase after pressing, with some material penetrating larger cavities alongside the glueline, the bonding of lignin is presumably mainly caused by interlocking of the rather randomly branched lignosulfonate aggregates. Thermoplastic fusion to afford a homogeneous phase is less likely due to the relatively high glass transition temperature of lignin (100–170 °C, [51]). Further aspects have to be considered as well. First, it is to recall that the solid content of LS-p was 15 wt.% only due to viscosity and processing constraints, thus accounting for less than one third of the solid content of white glue (approximately 50 wt.%). The significantly smaller particle size of lignin aggregates—on a rough estimate differing from PVAc latex particles by about two orders of magnitude [52]—along with their relatively good water-solubility; let us assume that this adhesive can more easily penetrate smaller cavities of wood. However, compared to the D3-class white glue, which contains diisocyanate as a cross-linker, further bonding of infused LS-p to the principal wood constituents is unlikely. In particular from that point of view, the tensile shear strength data of Figure 5 are intriguing, since they show that the novel LS-p adhesive and the conventional PVAc based adhesive share comparable mechanical properties, even though wood failure was significantly lower for LS-p.

A critical review of the results of all tensile shear strength measurements reveals significant inhomogeneities (cf. Figure 4 and Figure 5) within repetitions, albeit similar processing conditions have been employed. This refers first to the reproducibility of the pre-polymerization process, which turned out to be the most critical process step, as evident from the observed variations in molecular weight characteristics. Even though pre-polymerization was always only stopped after reaching a pre-defined viscosity level, the time required to reach these target values varied considerably due to both intentional and difficult to avoid variations within the set of parameters taking influence. The latter include the temperature, pH, enzyme activity and concentration, solid content of the formulation, presence of plasticizers, and the oxygen supply rate. Improved control of pre-polymerization will, therefore, be a central step on the way to an up-scaled production process. The lag time between pre-polymerization of the lignosulfonate and the actual application of the resulting adhesive including subsequent room-temperature pressing is another issue that requires harmonization. For the experiments conducted, it was assumed that polymerization would come to a standstill soon after stopping the oxygen supply, which, however, was not possible to monitor.

## 4. Conclusions

This study shows that an adhesive featuring similar tensile shear strength properties, such as a commercial D3 white glue, can be produced solely from magnesium lignosulfonate, which thus represents a 100% bio-based wood adhesive. The only pre-requisite that needs to be fulfilled is that the lignin is subjected to a laccase-mediated pre-polymerization process beforehand, using oxygen as a co-substrate. The significantly thinner glueline for the LS-p based adhesives which is obviously the reason for the low percentage of wood failure in LS-p bonded beech lamellas under tensile shear force literally requests for further optimization. This mainly includes the search for appropriate additives including cross-linkers rather than extending polymerization, since it was found that the impact of pre-polymerization and degree of polymerization, respectively, on the final bonding strength levelled-off after 30 min reaction time. The combination with appropriate natural softening and cross-linking agents would not only fully maintain the concept of a fully bio-based concept and green chemistry approach but would possibly also allow for a more convenient spreading of the glue, shorter pressing time and increased wood failure probability. With regard to upscaling, it would be important to ensure that lignosulfonates of the same type (e.g., magnesium lignosulfonate), derived from the same wood species (e.g., spruce), and obtained by the same pulping/separation/purification conditions are used. It is important since various properties including molecular structure, functional groups, degree of polymerization and content of further inorganic and organic constituents can vary to a larger extent. Finally, it is worth noting that laccase-mediated onward-polymerized magnesium lignosulfonate could be potentially used as a water-based primer for wood surfaces.

## Figures and Tables

**Table 1:** Descriptive statistics with mean values and standard deviation of viscosity (mPas) and tensile shear strength (N/mm^2^) of wood samples bonded with lignosulfonate-based adhesives (LS-p) without plasticizer (0P) or containing 5% glycerol (5G) or xylitol (5X). The degree of pre-polymerization was examined at four stages (0–3), where 0 was the reference without pre-polymerization.

	Pre-Polymerization		Viscosity (mPas)		Tensile Shear Strength (N/mm^2^)		
Plasticizer	Degree	Time (min)	Mean	SD	Mean	SD	n
0P	0	0	27.69	1.08	5.04	1.66	11
	1	25	181.20	27.04	8.65	1.61	15
	2	45	356.32	99.20	8.10	1.19	15
	3	60	707.55	42.10	7.91	1.77	14
5G	0	0	30.49	8.76	3.77	1.12	12
	1	30	95.40	13.87	8.29	1.40	15
	2	50	285.06	97.19	8.96	0.72	14
	3	110	562.68	134.66	8.31	1.06	12
5X	0	0	31.05	6.37	7.11	2.03	13
	1	30	55.65	9.95	8.89	0.90	15
	2	50	294.35	117.11	10.54	1.36	15
	3	110	395.32	144.73	9.46	1.92	12

**Table 2:** Descriptive statistics with mean values and standard deviation of tensile shear strength (N/mm^2^) of single-lap shear samples, bonded with lignosulfonate-based adhesives (LS-p) without plasticizer (0P), containing 5% glycerol (5G) or xylitol (5X) as plasticizers, compared to PVAc, *n* = 15.

	Tensile Shear Strength (N/mm^2^)	
Plasticizer	Mean Values	Std. Deviation
PVAc	10.96	1.07
LS-p 0P	10.57	1.42
LS-p 5X	10.54	1.36
LS-p 5G	8.96	0.72

## Data Availability

The data presented in this study are available on request from the corresponding author.
